# Development of Elvitegravir Resistance and Linkage of Integrase Inhibitor Mutations with Protease and Reverse Transcriptase Resistance Mutations

**DOI:** 10.1371/journal.pone.0040514

**Published:** 2012-07-18

**Authors:** Mark A. Winters, Robert M. Lloyd, Robert W. Shafer, Michael J. Kozal, Michael D. Miller, Mark Holodniy

**Affiliations:** 1 AIDS Research Center, Veterans Affairs Palo Alto Health Care System, Palo Alto, California, United States of America; 2 Division of Infectious Diseases and Geographic Medicine, Stanford University, Stanford, California, United States of America; 3 Research ThinkTank, Inc., Buford, Georgia, United States of America; 4 Yale University School of Medicine, New Haven, Connecticut, United States of America; 5 Veterans Affairs Connecticut Healthcare System, New Haven, Connecticut, United States of America; 6 Gilead Sciences, Foster City, California, United States of America; Burnet Institute, Australia

## Abstract

Failure of antiretroviral regimens containing elvitegravir (EVG) and raltegravir (RAL) can result in the appearance of integrase inhibitor (INI) drug-resistance mutations (DRMs). While several INI DRMs have been identified, the evolution of EVG DRMs and the linkage of these DRMs with protease inhibitor (PI) and reverse transcriptase inhibitor (RTI) DRMs have not been studied at the clonal level. We examined the development of INI DRMs in 10 patients failing EVG-containing regimens over time, and the linkage of INI DRMs with PI and RTI DRMs in these patients plus 6 RAL-treated patients. A one-step RT-nested PCR protocol was used to generate a 2.7 kB amplicon that included the PR, RT, and IN coding region, and standard cloning and sequencing techniques were used to determine DRMs in 1,277 clones (mean 21 clones per time point). Results showed all patients had multiple PI, NRTI, and/or NNRTI DRMs at baseline, but no primary INI DRM. EVG-treated patients developed from 2 to 6 strains with different primary INI DRMs as early as 2 weeks after initiation of treatment, predominantly as single mutations. The prevalence of these strains fluctuated and new strains, and/or strains with new combinations of INI DRMs, developed over time. Final failure samples (weeks 14 to 48) typically showed a dominant strain with multiple mutations or N155H alone. Single N155H or multiple mutations were also observed in RAL-treated patients at virologic failure. All patient strains showed evidence of INI DRM co-located with single or multiple PI and/or RTI DRMs on the same viral strand. Our study shows that EVG treatment can select for a number of distinct INI-resistant strains whose prevalence fluctuates over time. Continued appearance of new INI DRMs after initial INI failure suggests a potent, highly dynamic selection of INI resistant strains that is unaffected by co-location with PI and RTI DRMs.

## Introduction

HIV-1 integrase inhibitors (INI) are a relatively new class of antiretroviral (ARV) medications that function by preventing strand displacement and integration of the HIV-1 provirus into the host cell genome [1]. Raltegravir (RAL) was the first US FDA approved INI, and has demonstrated significant antiviral activity in ARV experienced and naïve patients when combined with other ARV classes [2], [3]. Elvitegravir (EVG) and dolutegravir (DTG) are INIs in clinical development and demonstrate comparable virologic activity in clinical trials [4]–[7].

Although INI drug-resistant mutations (DRMs) have rarely been described in ARV naïve, or INI naïve ARV experienced patients using conventional technologies [8], [9], virologic failure on INI-containing regimens has been described, and DRMs in the HIV-1 integrase (IN) coding region conferring phenotypic loss of susceptibility to these agents has been documented and reviewed elsewhere [10]. However, some IN DRMs confer resistance to several INIs (e.g., Q148HRK reduces RAL, EVG, and DTG susceptibility), others to some but not all INIs (e.g. N155H reduces susceptibility to RAL and EVG, but not DTG), and more than one pathway leading to INI resistance has been described (e.g., N155H, Q148HRK, or Y143RC for RAL resistance) [11]. Mutant strains have also been described *in vivo* from clinical isolates and by site directed mutagenesis where multiple DRMs on the same virus strand (N155H + E92Q), or addition of accessory mutations (Q148H + G140S), result in significantly greater loss of susceptibility [10]. In addition, certain INI DRMs result in a loss of viral fitness or replication capacity [12]–[14], and disappearance of INI DRMs after RAL discontinuation with resultant increase in RC has been described [15], [16], thus demonstrating the dynamic nature and complexity of INI resistance development.

Current commercial genotypic resistance assays generally use population sequencing to identify resistance to HIV-1 reverse transcriptase (RT), protease (PR) inhibitors and INIs by generating at least two separate amplicons (one for PR-RT, and one for IN). These assays cannot determine whether several INI DRMs occur on the same viral strand, evolve independently, or are present at low frequencies. Newer technologies, such as next generation sequencing (NGS) or parallel allele-specific sequencing (PASS), improve on the sensitivity of population sequencing by being able to detect low frequency variants in INI naïve and experienced patients [17], [18]. However, these assays cannot establish linkage between integrase inhibitor (INI), reverse transcriptase inhibitor (RTI), and protease inhibitor (PI) DRM because of the technical challenges of this analysis due to the length of sequence that must be interrogated. It is thus desirable to have a single amplification/amplicon generated during RT-PCR that can be used “universally” to genotype newer HIV-1 *pol* gene targets (e.g. RNase H or connection domain) as well as to understand the co-linkage and evolution of DRMs, and multiple polymorphisms and their role on resistance pathways among the three target functional enzymes.

Although significant work has described RAL-associated virologic failure and resistance development, less is known clinically about EVG resistance. Further, it is not clear whether INI DRM occur on the same viral strand (or viral quasispecies) as RTI and PI DRM. We analyzed amplicons covering the PR through IN coding region to determine whether HIV-1 INI DRM exist on quasispecies carrying PI and/or RTI DRM (co-linkage) and whether co-linkage between INI, PI, and/or RTI DRM differ among quasispecies or among INI mutational pathways in patients who have failed INI-containing ARV regimens.

## Results

**Figure 1:**
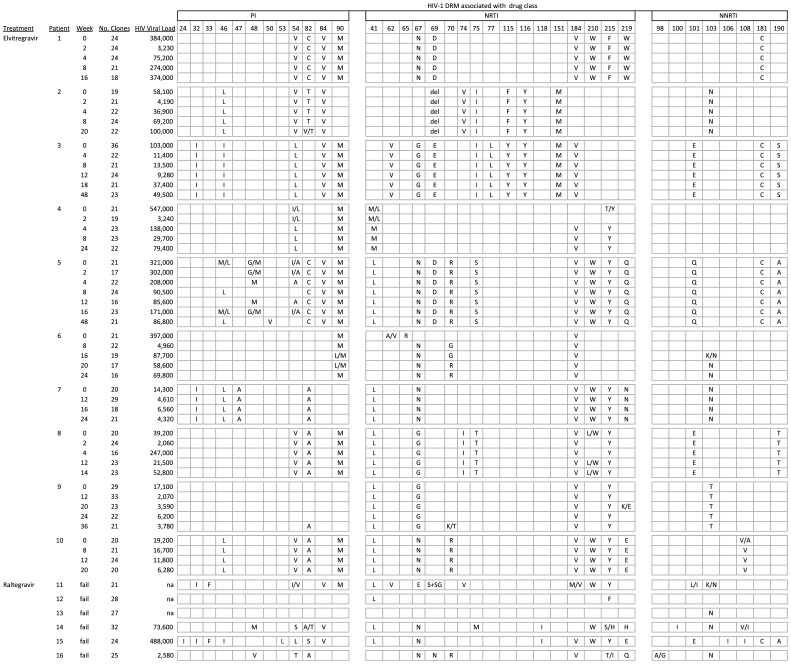
Protease and reverse transcriptase inhibitorDRM in HIV-1 strains from patients studied. Each cell shows the predominant DRM found at specific codons associated with resistance to PI, NRTI, and NNRTI for each patient’s HIV-1 strains at each time point. Two amino acids in a cell indicate the presence of a mixture of two mutant strains, or a mixture of wildtype and mutant strains. Mixtures were indicated when a mutation was found in at least 20% of the clones. “del” indicates a codon 69 deletion; “S+SG” indicates a codon 69 insert. “na”  =  not available. “fail” indicates time points collected after treatment failure in patients studied from standard clinical practice.

Ten EVG-treated patients and 6 RAL-treated patients were studied (Figure 1). Because the EVG-treated patients were part of a clinical trial, samples from serial time points were available for analysis. Approximately 5 time points per patient, ranging from 2 to 48 weeks of EVG treatment, were analyzed, and an average of 21 clones per time point (1120 total EVG clones) were generated. Only single, failure time points were available for the RAL-treated patients, and an average of 26 clones were analyzed from this group (157 total RAL clones).

### PCR-mediated Recombination

Mixtures of patient-derived plasmid clones were prepared, amplified, cloned, and approximately 30 clones per mixture were analyzed for the frequency of PCR-mediated recombination. PHI tests and Simplot analysis showed that among the 8 plasmid mixes tested, 4 mixes showed no significant recombination in the 664-bp IN coding region (p>0.05), while the 4 mixes that did show significant evidence of recombination (p<0.05) had only 1–2 recombinant clones per sample, producing an overall average of 0.75 recombination events per sample. When analyzing recombination across the PR/RT coding region, 3 of 8 plasmid mixes showed significant evidence (p<0.05) of recombination (1–3 recombinant clones per sample), with an overall average of 0.88 recombinants per sample. To investigate the frequency of recombination between the PR/RT and IN coding regions, the clone sequences (PR/RT and IN) were concatenated, and recombination analysis performed. Results showed that there was an average of 3 recombination events per patient (from a mean of 30 clones) that occurred between the RT and IN coding regions.

### PR and RT Mutations

Figure 1 shows the consensus PI, NRTI, and NNRTI DRM profile for each patient studied. All patients were infected with HIV-1 clade B strains. Because of the high degree of ARV experience of the patients studied, the PI and RTI DRM profiles were highly homogeneous (mean = 92%, range 40%–100%) within each patient’s clones at each time point evaluated. Most patient’s HIV strains exhibited DRM profiles consistent with high-level resistance to at least 2 drug classes. The EVG-treated patients had an average of 3.3 PI (range 0–6), 5.9 NRTI (range 2–9), and 1.3 NNRTI (range 0–3) DRM at baseline. Initial background regimens (BR) were limited to NRTIs and enfuvirtide in this study and, in general, the baseline PR and RT mutational profile was maintained with only some minor fluctuations throughout the follow-up period. At the last time point evaluated, 95.2% (101/106) PRI and RTI DRM found at baseline were still present, and the EVG-treated patients had an average of 3.3 PI, 5.1 NRTI, and 1.4 NNRTI resistance mutations. One patient developed a new PR mutation during EVG+BR treatment, 3 patients developed new NRTI mutations, and one patient developed a new NNRTI mutation compared to baseline. Considering subjects were not taking NNRTIs or PIs in their regimen, the PI and NNRTI mutations that developed likely reflect the recurrence of archived resistant viruses from previous treatments.

The RAL-treated patients had similar resistance profiles at failure compared to the EVG-treated patients at failure, with an average of 3.3 PI (range 0–8), 5.0 NRTI (range 0–8), and 2.2 NNRTI (range 0–5) resistance mutations.

### Mutations after EVG Treatment

**Figure 2:**
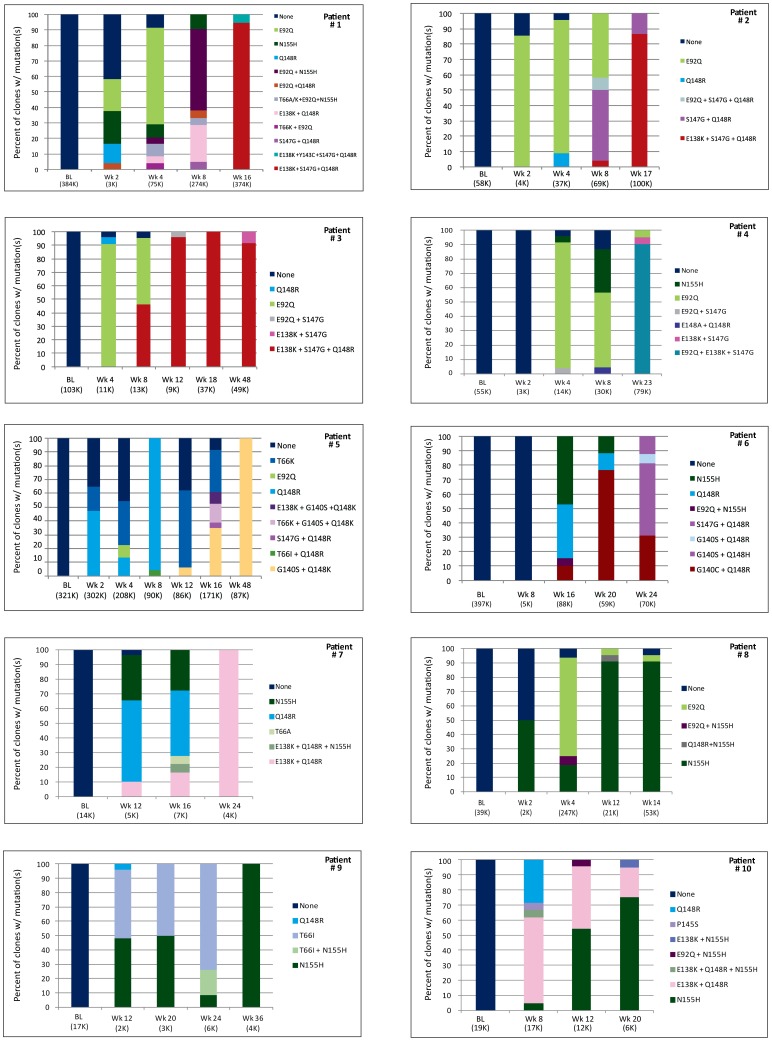
Distribution of HIV-1 integrase inhibitor resistance genotypes in patients treated with elvitegravir. Each bar represents the percentage of clones possessing the INI DRMs displayed in each legend. Values under each bar represent the week of EVG treatment, with the plasma HIV-1 viral load (K =  ×1000). Colors assigned each mutation or pattern are consistent across graphs.

Figure 2 shows the distribution of INI DRM found in the clones from EVG-treated patients at various time points. Since HIV viral loads in our patient samples were (>3,000 RNA copies/ml, sampling errors during the RT-PCR process were less likely to occur [19], [20]. In addition, in our study some samples with low viral loads had several INI-resistant quasi species, while some samples with high viral loads had few INI-resistant quasi species. Overall, our results showed that there was no relationship between the number of INI-resistant quasispecies and viral load (p = 0.15).

No major INI DRM were found in the baseline samples; however, strains with different INI resistance genotypes (i.e. single or combinations of INI DRM) were identified in the plasma of EVG-treated patients as early as 2 weeks after initiation of treatment. In the 5 patients that were analyzed at 2 weeks post treatment, an average of 1.6 strains carrying different INI resistance genotypes (range 0–4) were detected. The genotypes found at the early time points were primarily single mutations (T66A/K, E92Q, Q148R, and N155H), and several patients had more than one distinct population of single mutation-containing strains. At Week 4, patients (n = 6) had an average of 3.2 (range 2–6) different INI resistance genotypes, and this average was maintained throughout the later time points until week 24 and beyond, where the average number of INI resistant genotypes was 1.8 strains (range 1–4). These later time points showed both the influx of additional single mutation-containing strains, and the appearance of multiple distinct strains carrying 2 or more INI resistance mutations. The last time points studied in most patients showed the emergence of a dominant 2 or 3 mutation-containing strain (e.g. E138K + S147G + Q148R); although in 3 patients the dominant strain carried a single mutation, N155H.

### Intra-IN Coding Region Linkage

**Figure 3:**
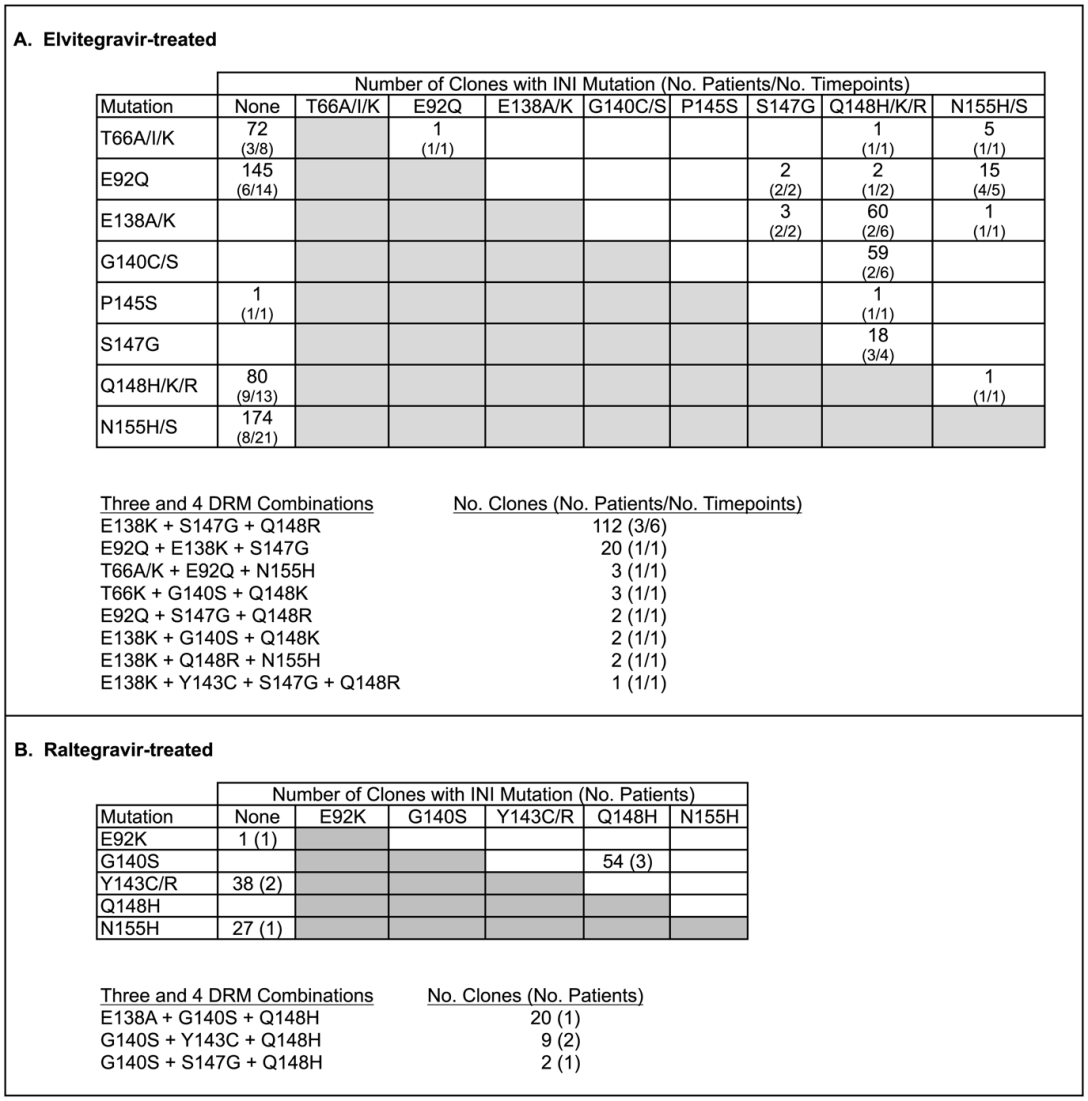
Frequency of INI DRM found in HIV-1 strains from patients treated with elvitegravir (A) or raltegravir (B). Occurrence of clones with single INI DRM are shown in the “none” column, and clones with two INI DRM are shown in the white cells. Strains with 3 or 4 INI DRM are shown in the bottom of each panel.

Figure 3 shows the co-existence of specific INI DRM on individual HIV clones in both EVG and RAL-treated patients. The bias of selecting more early treatment time points resulted in the highest frequency of strains containing single INI DRM; however some mutations were never found alone (e.g. E138A/K, G140C/S, and S147G, p<0.001). Several combinations of mutations were prevalent and found in multiple time points from multiple patients. In EVG-treated patients, the most prevalent two-mutation combinations on the same genome were G140C/S + Q148H/K/R, E138A/K + Q148H/K/R, S147G + Q148H/K/R, and E92Q + N155H/S. The most frequently occurring three-mutation combination was E138K+S147G+Q148R, found in 112 clones in 6 different time points from 3 patients. In contrast, there were no clones found that contained N155H/S together with either S147G or G140C/S.

In the more limited analysis of 5 RAL-treated patients, the most frequent single INI DRM-containing strains were N155H, also commonly seen in EVG-treated patients, and Y143C/R, which was not found in the EVG-treated patients. The most common strains containing more than one INI DRM were G140S + Q148H, with or without the additional mutations E138A or Y143C, observed in 3 patients.

### IN Mutation Linkage with PR and RT Mutations

The existence of positive or negative association of IN mutations with PR and/or RT mutations was examined in sequences from both EVG and RAL-treated patients. Table 1 shows the 8 most common INI DRM patterns and their associated PI and RTI DRM. Single or combinations of INI DRMwere found on strains carrying 0–6 PI mutations, 1–10 NRTI mutations, and/or 0–3 NNRTI mutations. This observation is consistent with results shown in Figure 2, where multiple different IN mutations evolved on the highly PR/RT mutated strains found at baseline in the EVG-treated patients. These data suggest that development of IN resistance mutations is not restricted by PR and/or RT resistance genotypes in highly ARV-experienced patients carrying multiple PI and RTI DRM.

**Table 1:** Linkage of the 8 most common INI DRM patterns to PR/RT DRM.

	Drug resistance genotypes associated with IN mutation(s)		
IN Mutation(s)	PI	NRTI	NNRTI
T66I/A/K	none	M41L, D67G, M184V, L210W, T215Y	K103N, K238T
	V32I, M46L, I47A, V82A	M41L, D67N, M184V, L210W, T215Y, +/−K219R	K103N
	G48M, I54A, V82C, I84V, L90M	M41L, D67N, T69D, K70R, L74V, V75S, M184V, L210W, T215F, K219Q	K101Q, Y181C, G190A
	M46L, V82C, I84V, L90M	M41L, D67N, T69D, K70R, L74V, V75S, M184V, L210W, T215F, K219Q	K101Q, Y181C, G190A
E92Q	I54V, V82A, L90M	M41L, D67G, L74I, V75T, M184V, +/− L210W, T215Y	K101E, G190T
	I54L, L90M	M41L, M184V, T215Y	none
	V32I, M46I, I54L, I84V, +/−N88S, L90M	A62V, D67G, K70E, V75I, F77L, Y115F, F116Y, Q151M, M184V	K101E, Y181C, G190S
	M46L, I54V, V82T, I84V	K70d, L74V, V75I, Y115F, F116Y, Q151M	K103N
	I54V, V82C, I84V, L90M	D67N, T69D, M184V, L210W, T215F, K219W	Y181C
	G48M, I54A, V82C, I84V, L90M	M41L, D67N, T69D, K70R, L74V, V75S, M184V, L210W, T215F, K219Q	K101Q, Y181C, G190A
	M46L, V82C, I84V, L90M	M41L, D67N, T69D, K70R, L74V, V75S, M184V, L210W, T215F, K219Q	K101Q, Y181C, G190A
Q148R	V32I, M46I, I54L, I84V, +/−N88S, L90M	A62V, D67G, K70E, V75I, F77L, Y115F, F116Y, Q151M, M184V	K101E, Y181C, G190S
	M46L, I54V, V82T, I84V	K70d, L74V, V75I, Y115F, F116Y, Q151M	K103N
	I54V, V82C, I84V, L90M	D67N, T69D, M184V, L210W, T215F, K219W	Y181C
	none	M41L, D67G, M184V, L210W, T215Y	K103N, K238T
	V32I, M46L, I47A, V82A	M41L, D67N, M184V, L210W, T215Y, +/−K219R	K103N
	L90M	D67N, K70G, M184V	K103N
	none	M184V	none
	G48M, I54A, V82C, I84V, L90M	M41L, D67N, T69D, K70R, L74V, V75S, M184V, L210W, T215F, K219Q	K101Q, Y181C, G190A
	M46L, V82C, I84V, L90M	M41L, D67N, T69D, K70R, L74V, V75S, M184V, L210W, T215F, K219Q	K101Q, Y181C, G190A
	M46L, I54V, V82A, L90M	M41L, D67N, K70R, M184V, L210W, T215Y, K219E	V108I
N155H	I54V, V82A, L90M	M41L, D67G, L74I, V75T, M184V, +/− L210W, T215Y	K101E, G190T
	V32I, M46L, I47A, V82A	M41L, D67N, M184V, L210W, T215Y, +/−K219R	K103N
	I54L, L90M	M41L, M184V, T215Y	none
	I54V, V82C, I84V, L90M	D67N, T69D, M184V, L210W, T215F, K219W	Y181C
	none	M41L, D67G, M184V, L210W, T215Y, +/− K219E	K103N, K238T
	V82A	M41L, D67G, M184V, L210W, T215Y, +/− K219E	K103N, K238T
	I84V	M41L, D67G, M184V, L210W, T215Y, +/− K219E	K103N, K238T
	L90M	D67N, K70G, M184V	none
	none	D67N, K70G, M184V	none
	L90M	D67N, K70G, M184V	K103N
	M46L, I54V, V82A, L90M	M41L, D67N, K70R, M184V, L210W, T215Y, K219E	V108I
	M46L, I54V, V82A, L90M	M41L, D67N, K70R, M184V, L210W, T215Y, K219E	none
E92Q + N155H	I54V, V82A, L90M	M41L, D67G, L74I, V75T, M184V, +/− L210W, T215Y	K101E, G190T
	I54V, V82C, I84V, L90M	D67N, T69D, M184V, L210W, T215F, K219W	Y181C
	L90M	D67N, K70G, M184V	none
	M46L, I54V, V82A, L90M	M41L, D67N, K70R, M184V, L210W, T215Y, K219E	none
E138K + Q148R	V32I, M46L, I47A, V82A	M41L, D67N, M184V, L210W, T215Y, +/−K219R	K103N
	I54L, L90M	M41L, M184V, T215Y	none
	M46L, I54V, V82A, L90M	M41L, D67N, K70R, M184V, L210W, T215Y, K219E	V108I
	M46L, I54V, V82A, L90M	M41L, D67N, K70R, M184V, L210W, T215Y, K219E	none
G140C/S + Q148R/H/K	L90M	D67N, K70G, M184V	K103N
	none	D67N, K70G, M184V	K103N
	G48M, I54A, V82C, I84V, L90M	M41L, D67N, T69D, K70R, L74V, V75S, M184V, L210W, T215F, K219Q	K101Q, Y181C, G190A
	M46L, V82C, I84V, L90M	M41L, D67N, T69D, K70R, L74V, V75S, M184V, L210W, T215F, K219Q	K101Q, Y181C, G190A
S147G + Q148R	M46L, I54V, V82T, I84V	K70d, L74V, V75I, Y115F, F116Y, Q151M	K103N
	I54V, V82C, I84V, L90M	D67N, T69D, M184V, L210W, T215F, K219W	Y181C
	L90M	D67N, K70G, M184V	K103N
	M46L, V82C, I84V, L90M	M41L, D67N, T69D, K70R, L74V, V75S, M184V, L210W, T215F, K219Q	K101Q, Y181C, G190A

## Discussion

Previous studies have described the development of RAL associated DRMs as early as 2 weeks after starting or restarting RAL treatment [16], changes in DRM patterns or pathways over time (N155H »Y143CHR or Q148H) [21]–[23], co-location of multiple RAL DRMs (i.e., Q148R/N155H) on the same strand evolving from separate mutants [17], and co-location of similar RAL DRM strains in plasma and peripheral blood mononuclear cell (PBMC) HIV-1 proviral DNA [24]. Others have described the presence of low frequency RAL DRMs (<1%) in some patients prior to starting RAL using allele-specific or ultra-deep sequencing, although the impact of these mutations on treatment failure is unclear [18], [25].

Preliminary analysis of EVG resistance in Study GS-183-0105 using population-based sequencing found a diversity of IN DRM patterns and changes in patterns over time [26], [27]. Our current clonal analysis examines multiple sequential time points and suggests a significant evolutionary IN coding region dynamic occurs when patients fail an EVG-containing regimen. In the patients studied here, early virologic failure showed the presence of several clonal populations carrying different INI DRMs. All patients started with single DRM mutants (i.e., E92Q or N155H) that either disappeared, were maintained, or altered through the addition of other DRMs or changes in amino acid composition at a given DRM position (e.g. G140S to G140C) in later-appearing species. In most patients, continued EVG treatment resulted in the reduction in the number of different INI-resistant quasispecies, and the emergence of a predominant population containing multiple INI DRMs or the single N155H. Certain INI DRMs were only found in association with other INI DRM (e.g., S147G or G140C/S with Q148H/K/R), suggesting that they either improve replication capacity or further decrease susceptibility to INI, whereas other DRMs like E92Q or N155H/S were more likely to occur alone. Although Q148R/N155H mutants have been described occurring on the same species after RAL therapy using NGS [17], but not in a previous study using conventional cloning and sequencing [28], this combination was found in only one clone from one patient in our study Several EVG treated patients exhibited species with 3 INI DRM (E138K/S147G/Q148R, E92Q/E138K/S147G), one of which has been shown to be additive in the loss of susceptibility to EVG [26], [29].

Analysis of mutational linkage data from PCR-amplified samples can be complicated by PCR-mediated recombination events [30]–[32]. We employed modifications to standard PCR procedures that have been shown to reduce the frequency of recombination (39, 44). In addition, we performed recombination analysis experiments to assess the number of these events in our PCR system. Within the IN coding region, we found less than 1 recombination event among the clones tested for each patient time point. While the recombination frequency within the IN coding region is very low, it is possible that some of the rare IN DRM genotypes found in our study (Figure 3) were the result of PCR-mediated recombination. The low recombination frequency was similar within the PR/RT coding region analyzed. The frequency was higher when analyzing recombination between the PR/RT and IN coding regions, however, this did not affect our finding of linkage between PR/RT and IN DRM, as 92% of the PR/RT clones had the same PR/RT DRM across all time points and all patients, due to the extensive ARV experience of the patients. Thus in this study, the few recombination events between the PR/RT and IN regions would not change the PR/RT DRM genotypes associated with the various INI DRM genotypes. However, in studies where patients have less ARV experience and/or PR/RT DRM, recombination may affect linkage results that may benefit from other analysis procedures like single genome sequencing.

The early appearance of multiple INI DRM-containing quasispecies in EVG-treated patients is similar to that seen in patients initially receiving NNRTI treatment [33], [34]. In contrast, the early development of PI and NRTI resistance is typically characterized by the emergence of a single DRM-containing quasispecies that is followed by the sequential addition of other DRM to the original genotype [33], [35]–[38]. The similarities between INI and NNRTI DRM development, in contrast to that of PI and NRTI, may be related to the fact that INI and NNRTI are more potent drugs, exerting a significantly greater selective pressure on the viral population and evoking a wider range of DRM-containing strains. In addition, low resistance barriers to INI DRM can allow multiple distinct pathways to develop that may have differences in the level of resistance conferred and are, in part, mutually exclusive. Co-existent DRM quasispecies present during early failure subsequently resolve to predominant resistant variants (containing single or multiple INI DRM) that exhibit the best ability to replicate in the presence of ongoing EVG drug pressure.

Previously published data for RT describes the step-wise loss of susceptibility occurring through the evolution of intermediates (i.e., M184I/V) or sequential development of resistance (leading to Q151M), and that multiple RT mutations are likely to reside or co-locate on single quasispecies [39], [40]. In addition, studies have described the co-linkage of multiple RT and/or PR DRMs on the same viral strand [41], and the evolution and intermediates with resultant single or limited species containing multiple ARV class DRMs on the same strand with continued ARV exposure to a failing regimen [42]. Our patients were all heavily ARV treated, with multi-class resistance, as evidenced by the numerous background RTI and PI DRMs, which remained remarkably consistent over time, implying that the additional fitness pressure of the INI DRM could be accommodated on the MDR backbone. Specifically, although certain RTI and INI DRM affect replication capacity or viral fitness [12], [29] that did not preclude, for example, the development of M184V in RT coding region and N155H or Q148HK/R in IN coding region from co-locating on the same strand and being detectable over time, implying no lethal effect of this DRM co-linkage. RT mutations can affect replication capacity, but in limited studies they have not affected INI susceptibility [43].

Population or current ultradeep sequencing of samples from patients who failed RAL or EVG have not resolved whether prior NRTI/NNRTI/PI DRM-containing quasispecies acquire IN mutations associated with RAL or EVG resistance, or circulate separately as distinct species, because of the short PCR amplicons analyzed. We employed a population-based RT-PCR method under conditions to reduce PCR-mediated recombination to assess quasi species variability and linkage. While our method does not eliminate the potential for PCR-mediated recombination, the results from our study suggest that this effect is limited. Over long distances (e.g. between PR and IN) recombination is difficult to assess since most samples were homogeneous with respect to PI and RTI DRM. Within the IN coding region, we did not find combinations of INI DRM known to be exclusive (or antagonistic) in RAL-resistant strains in this study or others [11], [23], [29], [44], which would be expected if recombination occurred with high frequency. Although others have reported on the use of long range PCR for evaluation of multi-coding region relationships in HIV [45], [46], to our knowledge, ours is the first report of co-localization of these DRMs across 3 distinct pol gene coding regions. For the most part, INI DRMs were added to already complex multi-drug resistant (MDR) species, as the patients that were studied received EVG or RAL only after failing other ARV regimens and typically had dominant viral strains with PI, NRTI, and NNRTI DRMs.

Our study has several limitations. First, the study was limited to a small number of patients with significant prior ARV exposure, multiple pre-existing DRMs, and suboptimal response to the EVG-containing regimen. It is not known whether INI DRM evolution or linkage relationships with RT and PR are the same in ARV-naïve patients or patients failing their first or second ARV regimen, or in patients with an initial strong virologic response (e.g. to undetectable) and subsequent failure. We only determined DRM evolution with EVG and not RAL, so the presence and evolution of RAL DRM intermediates, as others have described, using our clonal analysis could be different than those previously reported. In addition, we selected patients with a likelihood, based on preliminary population-based sequencing results, of possessing multiple DRM-containing strains. It is possible that a greater percentage of patients in the overall EVG-treated population, compared to what was surveyed here, develop INI resistance and failure with a limited number of DRM-containing quasispecies (as was seen in some patients in this study). Secondly, we did not generate in vitro drug susceptibility or replication capacity data on the strains in this study, so it was not possible to discern whether changes in mutational profiles are driven by drug selective pressure or simply represent random and/or stochastic fluctuations of variants that are equally capable of replicating in the presence of EVG. We only analyzed patients with HIV-1 clade B virus, and so could not determine whether evolution or linkage is the same in non-clade B strains. Previous studies have indicated that RAL is equally efficacious in non-clade B virus infections and that DRMs that develop after RAL failure are similar to clade B strains [47], although novel DRMs that confer RAL resistance have also been described in circulating recombinant forms (G118R) and non-clade B strains [48], [49].

Finally, the number of clones analyzed may be insufficient to determine prevalence of additional very low frequency mutants, as has been described with NGS, even in patients without exposure to INIs. Previous studies have found that low frequency NNRTI mutants can result in higher level of virologic failure [50]. Although low frequency mutants with RAL DRMs have been found prior to RAL therapy, they have not affected virologic outcome in most of those patients studied [18]. We did not find major INI DRMs prior to EVG treatment, although it is possible that EVG mutants existed and lead to early virologic failure. Further analysis using NGS would need to be performed to answer this question. In addition, other quasispecies-probing techniques, such as single-genome sequencing, may yield different proportions of quasispecies at the various time points – our goal was not to accurately quantify the quasispecies populations, but to survey its possible breadth and evolution over time.

In summary, EVG ARV regimen failure demonstrates a dynamic evolution of multiple species during early failure leading to a final DRM associated species. EVG and RAL DRMs were co-located on the same viral strand as RT and PR DRMs. Co-linear genotypic analysis of long-range amplification products supports the utility for whole HIV-1 *pol* viral sequencing to provide a greater comprehensive resistance profile for use in guiding ARV treatments and prognosis.

## Materials and Methods

### Ethics Statement

The Yale University/VA Connecticut Institutional Review Board approved the study and written informed consent was obtained from all subjects studied at Yale. The Stanford University IRB approved the study with a waiver of consent, as some samples for this study were obtained at Stanford after routine clinical laboratory testing with safeguards in place for protection of personal health information. The Western IRB (Olympia, WA) and Chesapeake IRB (Columbia, MD) approved Gilead Study 183-0105, and written informed consent was obtained from all study subjects.

### Patients

Ten EVG-treated patients that were enrolled in Gilead 183-0105 [6], a dose-ranging phase 2-study that explored the use of ritonavir-boosted EVG in the absence of PIs with optimized RTI background in heavily treatment-experienced patients, were studied. A convenience sample of patients was selected from this study based on the following criteria: 1) received 125 mg/day of EVG; 2) had cryopreserved plasma from multiple time points; 3) had virologic failure on their EVG-containing regimen, and 4) had evidence of evolving EVG resistance (mixtures) based on preliminary population-based genotypes. In addition, plasma samples from 5 raltegravir-experienced patients were obtained from remnant material from clinical practice; only single, failure time points were available from these patients.

### Amplification and Clonal Analysis

RNA was isolated from 500 µl of plasma with Qiagen Viral MinElute Kits (Qiagen, Chatsworth, CA) and amplified by RT-nested PCR using conditions previously shown to reduce the frequency of PCR-mediated recombination [46], [51] . These conditions include using a mixture of rTth polymerase and the proofreading polymerase VentR, hot start, and long extension times in both the RT and PCR steps. Reverse transcription was performed using Superscript III First Strand Kits with random hexamers according to manufacturer’s instructions. The RT conditions were 25°C for 10 min, 45°C for 2 hr, and 85°C for 5 min. The resulting cDNA was amplified for 40 cycles with the GeneAmp XL PCR kit (Applied Biosystems, Foster City, CA), using primers MAW26 (TGG GAA ATG TGG AAA GGA AGG AC) and VIFR5 (GGG ATG TGT ACT TCT GAA CTT), which generates an amplicon from the protease coding region through the integrase coding region. Amplification parameters were 1 cycle of 94°C for 1 min, then 35 cycles of 94°C for 15 sec, 53°C for 15 sec, and 72°C for 10 min, and a final 10 min extension at 72°C. A second round PCR was performed under the same amplification conditions using primers PRO-1 (CAG AGC CAA CAG CCC CAC CA) and MAW24 (TGC TGT CCC TGT AAT AAA CCC GAA AAT). Limiting dilution analysis on a subset of samples used in this study showed that as few as 300 input HIV RNA copies could be successfully amplified using this method. The resulting 2.7 kb amplicons were cloned using TOPO TA cloning kits (Invitrogen, Carlsbad, CA) according to manufacturer’s instructions. Minipreps from the resulting clones were prepared using Qiagen Turbo96 Miniprep kits, and the sequences of codons 1–99 of PR, 1–230 of RT, and 1 - 219 of IN coding regions were determined using standard dideoxyterminator sequencing (Sequetech, Mountain View, CA) using primers M13F and M13R (for PR and IN) and primer RT20 (CTG CCA GTT CTA GCT CTG CTT C, for RT).

Standard phylogenetic analysis was performed to rule out contamination between patients. Consensus sequences were generated from alignment of the clone sequences using MegAlign (DNAstar, Madison, WI), and mixtures were reported if a minority population was represented in greater than 20% of the clones. Drug resistance mutations for PI, NRTI, NNRTI, and INI in each clone were identified by the Stanford Drug Resistance database [52]. Statistical analyses using inear regression,Chi-Square, and Fisher’s exact tests were performed using VassarStats (http://faculty.vassar.edu/lowry/VassarStats.html).

### Recombination Analysis

The frequency of PCR-mediated recombination in this study was determined by mixing equal proportions (1000 copies each) of unique patient-derived plasmid clones. For example, clone 11 from Patient 3 was mixed with clone 1 from patient 8. Eight separate plasmid mixtures were prepared and amplified as described above, except that the first round primers were M13F and M13R. The resulting second round amplicons (using primers PRO-1 and MAW24) were cloned and sequenced as described above. Sequences from approximately 30 clones per mixture were assembled, aligned, and tested for the presence of recombinant clones using the PHI test in the SplitsTree software package (www.splitstree.org) [53]. Alignments were further evaluated for recombination using SimPlot (http://sray.med.som.jhmi.edu/SCRoftware/simplot/) [54].

### Nucleotide Sequences

Sequences of all clones were submitted to Genbank under accession numbers JX198692 - JX202525.
